# Supplementary material for: Longitudinal symptom dynamics of COVID-19 infection
Source: Nat Commun. 2020 Dec 4;11:6208. doi: 10.1038/s41467-020-20053-y (PMC7718370; doi:10.1038/s41467-020-20053-y)
Supplement: Supplementary file 1 — Supplementary Information [file 41467_2020_20053_MOESM1_ESM.pdf]

Supplementary Information

Supplementary note 1: Cohort selection

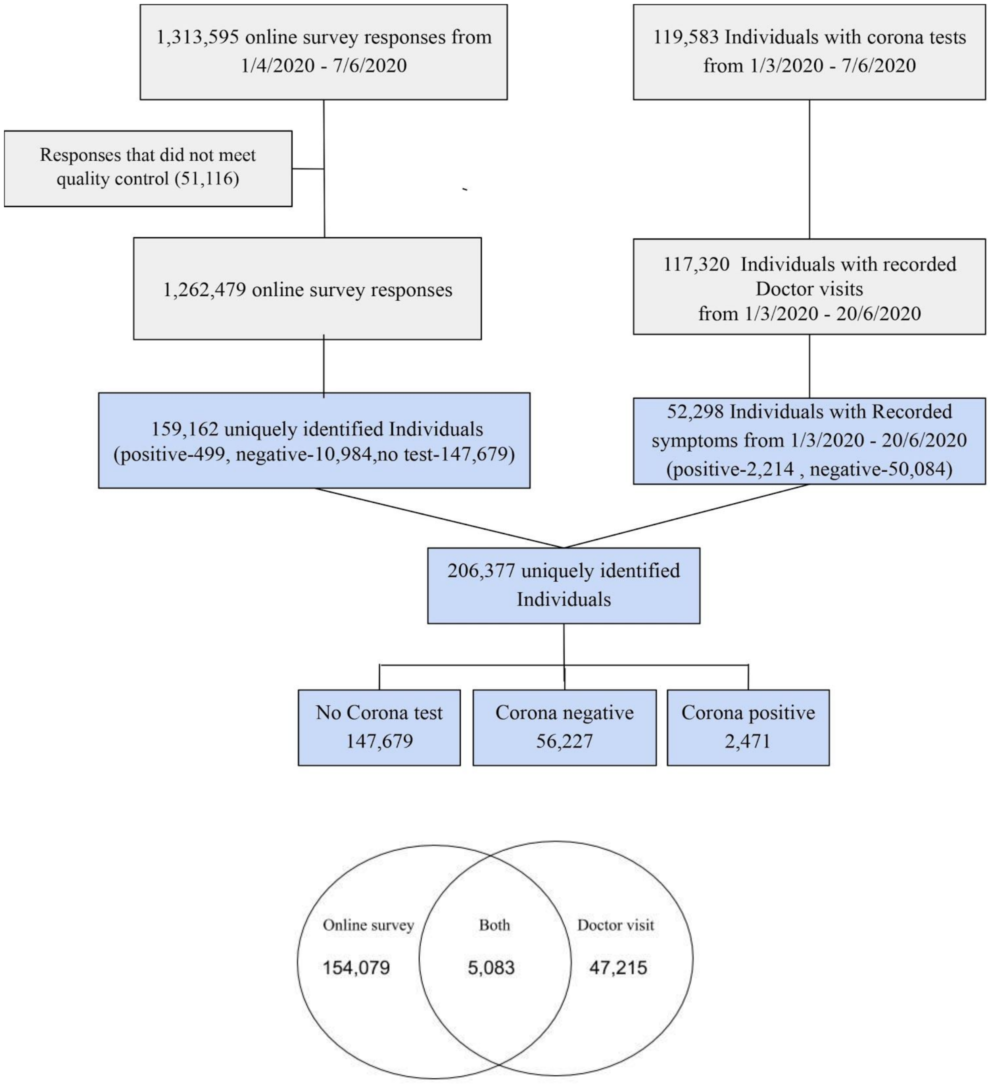

Supplementary Figure 1. Cohort selection A: A flow chart of cohort selection. In the process of quality control, surveys that were not fully completed by participants were excluded from the analysis. B: Venn diagram of the data sources from which information on symptoms were obtained. The number of individuals that had information on symptoms from the online survey, primary care visits recorded in the electronic health records and both data sources are presented.

Symptom	total	Symptom appears in both data sources	Symptom was only recorded in a primary care visit	Symptom was only self-reported in the survey
Cough	393	206 (52%)	70 (18%)	117 (30%)
Diarrhea	103	36 (35%)	20 (19%)	47 (46%)
Disturbances Of Sensation Of Smell And Taste	32	0 (0%)	5 (16%)	27 (84%)
Dyspnea and or Shortness of breath	116	5 (4%)	11 (9%)	100 (86%)
Fatigue	312	51 (16%)	44 (14%)	217 (70%)
Fever	165	56 (34%)	99 (60%)	10 (6%)
Headache	248	34 (14%)	19 (8%)	195 (79%)
Myalgia	311	34 (11%)	139 (45%)	138 (44%)
Nausea and or vomiting	63	2 (3%)	9 (14%)	52 (83%)
Runny nose and or nasal congestion	193	6 (3%)	8 (4%)	179 (93%)
Sore throat	202	18 (9%)	13 (6%)	171 (85%)

Supplementary Table 1: Comparison between self-reported symptoms and Electronic health record-captured data.

Supplementary note 2: COVID-19 survey

Age: _____

Gender:
- ❑ Male
- ❑ Female

I am:
- ❑ Feeling well
- ❑ Not feeling well

Are you experiencing any of the following symptoms?

Cough
- ❑ Dry cough (no sputum)
- ❑ Wet cough (with sputum)

- ❑ Fatigue
- ❑ Muscle pain
- ❑ Shortness of breath
- ❑ Rhinorrhea (runny nose) and/or nasal congestion
- ❑ Diarrea
- ❑ Nausea and/or vomiting
- ❑ Sore throat
- ❑ Headache
- ❑ Chills
- ❑ Confusion
- ❑ Loss of taste or smell
- ❑ Other symptoms- ____________

I am currently:
- ❑ Not in isolation
- ❑ In isolation (including from family members, staying in a separate room) from the date of _____ due to:
  - ❑ A recent international travel
  - ❑ A contact with an individual who was infected with coronavirus
  - ❑ Experiencing disease symptoms
  - ❑ Voluntary isolation

- ❑ I have a confirmed infection with COVID-19 (by a lab test) and currently:
  - ❑ In home isolation
  - ❑ Staying in a hotel
  - ❑ Hospitalized in a hospital
  - ❑ I recovered from COVID-19 infection

Cigarette smoking habits:
- ❑ I currently smoke
- ❑ I used to smoke and stopped more than 5 years ago
- ❑ I used to smoke and stopped less than 5 years ago
- ❑ I have never smoked

What is your current body temperature?

- ❏ I did not measure my temperature in the last 24 hours
- ❏ I measured my temperature (degrees celsius) and the highest value was :
  - ❏ Below 36 °C
  - ❏ 36-36.9 °C
  - ❏ 37-37.4 °C
  - ❏ 37.5-37.9 °C
  - ❏ 38-38.4 °C
  - ❏ 38.5-38.9 °C
  - ❏ 39-39.9 °C
  - ❏ Above 40 °C

How many individuals have you been in contact with in the last 24 hours?
(within approximately 2 meters (6 ft 7 in) for more than 15 minutes)
Adults (age above18 years old _____)
Children (age below 18 years old _____)

Do you work outside home?

- ❏ Yes- Did you meet with more than 10 people a day at work in the last two weeks ? Yes/No
- ❏ No

Do you, or anyone of your household members is a part of a medical team, actively treating patients?

- ❏ Yes
- ❏ No

Supplementary note 3: Extracting symptoms from EHR

Symptoms were extracted from the EHR using the relevant ICD-9 codes

Symptoms group	ICD-9 Codes
Disturbances in sensation of smell and taste	781.1 - Disturbances of sensation of smell and taste, Anosmia; Parageusia; Parosmia
Emotional disturbance	309.1 - Adjustment reaction with prolonged depressive reaction
	309.8 - Other specified Adjustment reactions
	308.0 - Predominant disturbance of emotions, Anxiety,Emotional crisis,Panic state as acute reaction to exceptional (gross) stress
	799.90 - Diagnosis or condition deferred on Axis 1

	300.00 - Anxiety state, unspecified Anxiety: neurosis, reaction, state (neurotic); Atypical anxiety disorder
	309.9 - Unspecified Adjustment reaction, Adaptation reaction NOS; Adjustment reaction NOS
	309.0 - Adjustment reaction with brief depressive reaction, Adjustment disorder with depressed mood; Grief reaction
	300.4 - Neurotic Depression Anxiety depression; Depression with anxiety; Depressive reaction; Dysthymic disorder; Neurotic depressive state; Reactive
	311 - Depressive disorder,Depressive disorder NOS; Depressive state NOS; Depression NOS
	V62.8 - Other Psychological or Physical stress
Tachycardia	785.0-Tachycardia NOS
Fever	780.60-Fever NOS
Nausea and/or vomiting	787.02 - Nausea alone Emesis
	787.0 - Nausea and vomiting
	787.03 - Vomiting alone Emesis
Chest pain or discomfort	786.59 - Other chest pain (discomfort,pressure, tightness) in chest
	786.50 - Unspecified chest pain
Fatigue	780.7 - Malaise and fatigue, Asthenia NOS, Lethargy; Postviral (asthenic) syndrome; Tiredness
Voice disturbance	784.49 - Other voice disturbance, change in voice; Dysphonia; Hoarseness; Hypernasality; Hyponasality
Syncope	780.2 - Syncope and collapse, Blackout; Fainting; (Near, Pre) syncope; Vasovagal attack
Sleep disturbance	780.52 - Other Insomnia, Insomnia NOS
	780.50-Sleep disturbance NOS
Headache	784.0 - Headache, Facial pain; Pain in head NOS
Runny nose and/or nasal congestion	460 - Acute Nasopharyngitis (common Cold), Coryza (acute); Nasal catarrh, acute; Nasopharyngitis: NOS, acute, infective NOS; Rhinitis: acute
Palpitation	785.1 - Palpitations awareness of heart beat
Rash	782.1 - Rash and other nonspecific skin eruption, Exanthem

Dyspnea and/or Shortness of breath	786.09 - Other Dyspnea and respiratory abnormality, Apnea; Cheyne-Stokes respiration; Respiratory: distress, insufficiency; Shortness of breath
Myalgia	729.5 - Pain In Limb
	729.1 - Myalgia And Myositis, Unspecified Fibromyositis NOS
	724.5 - Backache, Unspecified Vertebrogenic (pain) syndrome NOS
	723.1 - Cervicalgia, Pain in neck
	724.2 - Lumbago, Low back pain; Low back syndrome; Lumbalgia
Cough	786.2 - Cough
Arthralgia	719.46 - Pain in joint involving lower leg Arthralgia; Fibula; Knee joint; Patella; Tibia
	719.40 - Pain In Joint, Site Unspecified, Arthralgia
Dizziness	780.4 - Dizziness and Giddiness, Light-headedness; Vertigo NOS
Diarrhea	787.91 - Diarrhea, Diarrhea NOS
Speech disturbance	784.5 - Other speech disturbance, Dysarthria; Dysphasia; Slurred speech
Weight loss	783.2 - Abnormal loss of weight
Sore throat	784.1 - Throat pain
Conjunctivitis	372.0 - Acute conjunctivitis
General symptoms (Amnesia / Chills / Generalized pain /Hypothermia)	780.9 - Other general symptoms, Amnesia (retrograde); Chill(s) NOS; Generalized pain; Hypothermia, not associated with low environmental temperature
Abdominal pain	789.06 - Abdominal pain, epigastric abdominal tenderness; Colic: NOS, infantile; Cramps, abdominal; Epigastric pain; Umbilical pain
Lymphadenopathy	785.6 - Enlargement of lymph nodes, Lymphadenopathy; "Swollen glands"
Constipation	564.0 - Constipation
Disturbance of skin sensation	782.0 - Disturbance of skin sensation, Anesthesia of skin; Burning or prickling sensation; Hyperesthesia; Hypoesthesia; Numbness; Paresthesia; Tingling
Neuralgia	729.2 - Neuralgia, Neuritis, And Radiculitis, Unspecified

Supplementary Table 2: International Classification of Diseases,Ninth Revision (ICD-9) Codes used to define symptoms group. NOS- not otherwise specified.

Supplementary note 4: Symptoms and time of recovery analysis

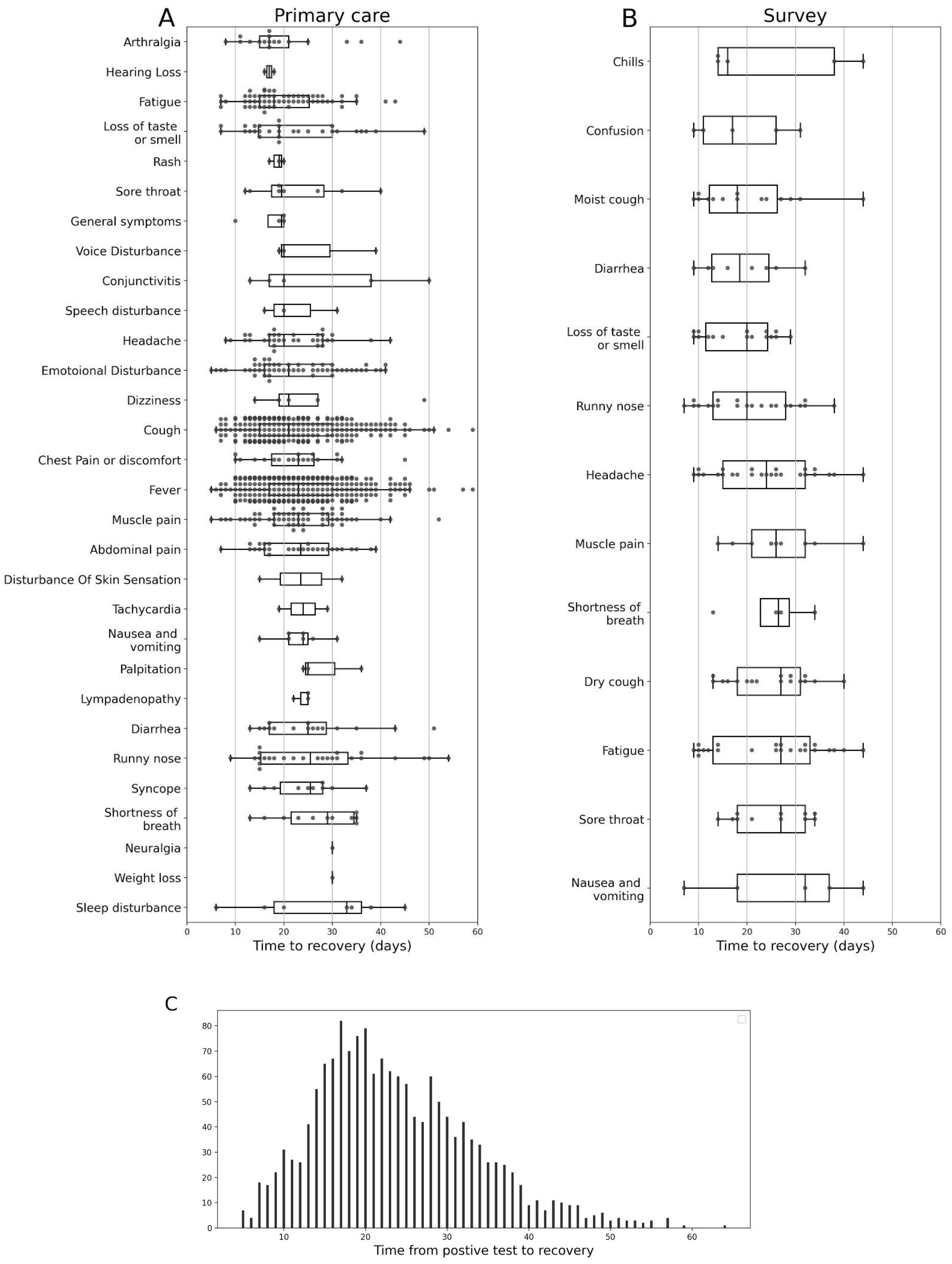

Supplementary Figure 2. Recovery time from COVID-19. A+B: each point indicates recovery time for a COVID-19 positive individual who experienced a specific symptom before or after testing positive. Boxplots show quartiles of the distribution of recovery times for each symptom considered. A primary care visits and B surveys C Histogram presenting the number of days from diagnosis to recovery in the cohort. Boxplots bounds are centered at the median value and bounds are drawn at the

interquartile range (IQR = Q3-Q1). Upper and lower whiskers extend to last datum less than Q3 ±whisker*IQR respectively. Beyond the whiskers, data are plotted as individual points.

Supplementary note 5: Associations between symptom reports and positive tests

A. self-reported symptoms in adults

self-reported symptoms in adults	Basic Model		Adjusted Model		IPW		IPW Adjusted	
	OR	CI	OR	CI	OR	CI	OR	CI
Loss of taste and smell	11.18	[6.43-19.44]	9.34	[5.26-16.58]	15.46	[6.02-39.74]	12.67	[4.79-33.46]
Confusion	4.02	[1.58-10.21]	3.14	[1.21-8.18]	4.44	[1.16-16.96]	3.71	[0.95-14.41]
Headache	2.03	[1.29-3.19]	1.83	[1.15-2.90]	2.08	[1.1-3.94]	1.91	[1-3.64]
Fatigue	1.73	[1.08-2.79]	1.72	[1.06-2.80]	1.85	[0.83-4.09]	1.83	[0.82-4.09]
Fever (Body temperature above 38°C)	1.58	[0.63-3.94]	1.48	[0.59-3.74]	2.13	[0.15-30.17]	2.05	[0.14-29.55]
Dry cough	1.52	[0.91-2.54]	1.18	[0.70-2.00]	1.68	[0.73-3.87]	1.34	[0.58-3.11]
Diarrhea	1.44	[0.69-3.00]	1.34	[0.64-2.83]	2.42	[0.96-6.15]	2.26	[0.88-5.76]
Rhinorrhea and/or nasal congestion	1.42	[0.88-2.29]	1.23	[0.76-1.99]	1.47	[0.79-2.75]	1.29	[0.68-2.42]
Cough	1.42	[0.89-2.25]	1.08	[0.67-1.72]	1.37	[0.68-2.77]	1.07	[0.53-2.18]
Nausea and/or vomiting	1.32	[0.53-3.28]	1.17	[0.46-2.95]	1.78	[0.43-7.44]	1.55	[0.37-6.54]
Wet cough	1.25	[0.69-2.26]	0.99	[0.54-1.81]	1.16	[0.44-3.1]	0.92	[0.34-2.46]
Chills	1.01	[0.41-2.52]	0.92	[0.37-2.30]	2.18	[0.46-10.42]	1.97	[0.41-9.52]
Myalgia	0.97	[0.50-1.88]	1	[0.51-1.95]	0.8	[0.22-2.98]	0.82	[0.22-3.05]
Shortness of breath	0.89	[0.36-2.20]	0.73	[0.29-1.83]	0.94	[0.18-4.86]	0.79	[0.15-4.12]
Other symptoms	0.71	[0.29-1.77]	0.9	[0.36-2.26]	0.91	[0.29-2.88]	1	[0.32-3.17]
Sore throat	0.69	[0.37-1.27]	0.65	[0.35-1.22]	0.63	[0.18-2.17]	0.59	[0.17-2.06]
Body temperature below 37.4 °C	0.53	[0.34-0.80]	0.68	[0.44-1.05]	0.55	[0.34-0.89]	0.66	[0.41-1.07]

No symptoms	0.34	[0.22-0.53]	0.51	[0.32-0.80]	0.43	[0.27-0.69]	0.53	[0.33-0.86]
Body temperature between 37.5°C and 37.9°C	0.33	[0.05-2.38]	0.36	[0.05-2.62]	0.22	[0-103.13]	0.22	[0-105.68]

Supplementary Table 3: Odds ratio calculation for self-reported symptoms in adults 21 days prior to the date of diagnosis. Adjusted model takes into account the following covariates : age, gender, presence of a chronic medical condition and time (number of days since study initiation).Inverse Probability weighting (IPW) was applied by fitting a logistic regression model for the probability of being tested (regardless of result) For positive COVID19 cases, this date was considered as the first positive PCR test. For COVID negative cases, this test was considered as the first negative result for COVID-19.

B. Symptoms documented in primary care in children

Primary care documented symptoms in children	Basic model		Adjusted Model	
	OR	CI	OR	CI
Disturbances Of Sensation Of Smell And Taste	2.45	[0.32-18.87]	2.26	[0.28-18.54]
Syncope	2.45	[0.58-10.40]	1.94	[0.42-8.86]
Emotional Disturbance	2.03	[1.03-4.02]	1.60	[0.79-3.24]
Sleep disturbance	1.47	[0.20-10.97]	1.84	[0.23-14.77]
Fatigue	1.39	[0.73-2.64]	1.33	[0.69-2.56]
Dizziness	1.18	[0.16-8.69]	1.00	[0.13-7.67]
Conjunctivitis	1.15	[0.42-3.14]	1.17	[0.42-3.26]
Rash	0.81	[0.30-2.21]	1.06	[0.38-2.93]
Sore throat	0.66	[0.24-1.79]	0.66	[0.24-1.81]
Myalgia	0.64	[0.20-2.02]	0.54	[0.17-1.72]
Arthralgia	0.61	[0.08-4.44]	0.58	[0.08-4.32]
Headache	0.55	[0.21-1.49]	0.39	[0.14-1.07]
Runny nose and or nasal congestion	0.47	[0.12-1.90]	0.53	[0.13-2.21]
Speech disturbance	0.45	[0.06-3.26]	0.57	[0.08-4.26]
Dyspnea and or Shortness of breath	0.43	[0.16-1.17]	0.50	[0.18-1.38]
Cough	0.40	[0.28-0.59]	0.36	[0.25-0.53]
Fever	0.30	[0.22-0.42]	0.34	[0.24-0.47]

Nausea and or vomiting	0.22	[0.03-1.60]	0.20	[0.03-1.42]
Diarrhea	0.17	[0.04-0.70]	0.22	[0.05-0.87]
Abdominal pain	0.10	[0.01-0.69]	0.09	[0.01-0.61]

Supplementary Table 4: Odds ratio calculation for symptoms in children that were documented in primary care visits 21 days prior to the date of diagnosis. Adjusted model takes into account the following covariates : age, gender, presence of a chronic medical condition and time (number of days since study initiation). For positive COVID19 cases, this date was considered as the first positive PCR test. For COVID negative cases, this test was considered as the first negative result for COVID-19.

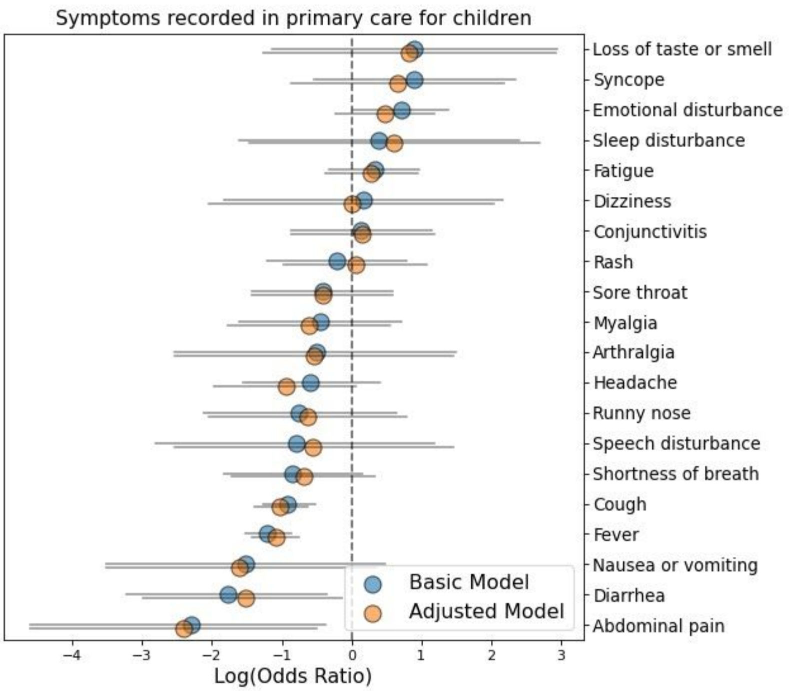

Supplementary Figure 3: Odds ratio calculation for symptoms in children that were documented in primary care visits 21 days prior to the date of diagnosis (n=17,736). Adjusted model takes into account the following covariates: age, gender, presence of a chronic medical condition and time (number of days since study initiation). Calculated log odds ratios are presented along with grey lines indicating 95% confidence intervals.

C. Primary care documented symptoms in adults

Primary care documented symptoms in adults	Basic model		Adjusted model	
	OR	CI	OR	CI
Disturbances of sensation of smell and taste	5.47	[3.69-8.09]	6.83	[4.51-10.33]
Syncope	2.09	[1.13-3.88]	2.52	[1.33-4.77]
Runny nose and or nasal congestion	2.09	[1.47-2.95]	1.9	[1.33-2.72]

Fever	1.62	[1.44-1.83]	1.66	[1.47-1.87]
Speech disturbance	1.37	[0.33-5.69]	1.17	[0.27-5.03]
Sleep disturbance	0.94	[0.48-1.84]	1.12	[0.57-2.21]
Fatigue	0.89	[0.69-1.14]	1.17	[0.91-1.52]
Cough	0.89	[0.78-1.01]	0.79	[0.69-0.90]
Headache	0.79	[0.56-1.12]	0.87	[0.61-1.24]
Nausea and or vomiting	0.76	[0.42-1.39]	0.82	[0.45-1.50]
Myalgia	0.69	[0.55-0.87]	0.76	[0.60-0.97]
Emotional disturbance	0.67	[0.52-0.86]	0.79	[0.61-1.01]
Dizziness	0.57	[0.30-1.07]	0.74	[0.39-1.39]
Abdominal pain	0.53	[0.38-0.75]	0.59	[0.42-0.83]
Dyspnea and or Shortness of breath	0.51	[0.26-0.98]	0.42	[0.22-0.82]
Diarrhea	0.46	[0.29-0.74]	0.69	[0.43-1.10]
Sore throat	0.35	[0.19-0.66]	0.44	[0.24-0.83]
Conjunctivitis	0.13	[0.02-0.95]	0.11	[0.02-0.77]

Supplementary Table 5: Odds ratio calculation for symptoms in Adults that were documented in primary care visits 21 days prior to the date of diagnosis. Adjusted model takes into account the following covariates : age, gender, presence of a chronic medical condition and time (number of days since study initiation). .For positive COVID19 cases, this date was considered as the first positive PCR test. For COVID negative cases, this test was considered as the first negative result for COVID-19.

Supplementary note 6: Hazard ratios analysis

1. Self-reported symptoms among individuals who were tested for COVID-19 in time. Outcome was considered the first positive PCR test for COVID-19. Negative test results or time after 21 days was censored.

self - reported symptoms	All surveys		Surveys weighted	
	HR	95% CI	HR	95% CI
Loss of taste and smell	22.49	[18.95-26.70]	13.44	[10.52-17.17]
Fever (body temperature above	9.75	[6.07-15.66]	4.57	[2.36-8.85]

38°C)				
Confusion	5.43	[4.00-7.36]	5.12	[3.38-7.77]
Headache	2.6	[2.26-3.00]	2.85	[2.36-3.44]
Dry cough	2.6	[2.23-3.03]	2.02	[1.63-2.52]
Diarrhea	2.54	[2.00-3.22]	3.74	[2.81-4.99]
Fatigue	2.44	[2.04-2.92]	3.03	[2.43-3.78]
Other symptoms	2.3	[1.89-2.80]	1.4	[0.96-2.03]
Chills	2.26	[1.50-3.40]	3.11	[2.02-4.77]
Myalgia	2.01	[1.62-2.50]	1.42	[0.99-2.05]
Rhinorrhea and/or nasal congestion	1.88	[1.66-2.13]	1.59	[1.31-1.93]
cough	1.63	[1.42-1.87]	1.53	[1.26-1.85]
Nausea and/or vomiting	1.61	[1.08-2.40]	2.76	[1.84-4.13]
Sore throat	1.24	[1.00-1.55]	0.86	[0.61-1.22]
Body temperature below 37.4 °C	1.02	[0.93-1.11]	0.82	[0.71-0.95]
Shortness of breath	0.89	[0.60-1.34]	0.56	[0.29-1.06]
Wet cough	0.86	[0.68-1.08]	1.04	[0.79-1.38]
Body temperature between 37.5°C and 37.9°C	0.64	[0.17-2.43]	0.77	[0.2-2.93]
No symptoms	0.48	[0.44-0.52]	0.42	[0.37-0.49]

Supplementary Table 6: Hazard ratios for each of the self-reported symptoms, calculated by Cox proportional hazards models, and adjusted for age, gender, presence of a chronic medical condition and time (number of days since study initiation) are presented to account for the time from symptoms onset to COVID-19 testing results.

2. Symptoms documented on EHR among individuals who tested for COVID-19 in time. Outcome considered was the first positive PCR test for COVID-19

Symptom recorded on EHR	All records		Weighted	
	HR	95% CI	HR	95% CI

Disturbances Of Sensation Of Smell And Taste	13.29	[9.56-18.47]	12.17	[8.43-17.57]
Fever	4.32	[3.80-4.90]	4.34	[3.74-5.04]
Fatigue	1.76	[1.41-2.19]	1.81	[1.39-2.35]
Syncope	1.72	[0.99-2.96]	1.95	[1.04-3.66]
Cough	1.42	[1.25-1.61]	1.45	[1.25-1.68]
Runny nose and or nasal congestion	1.38	[1.01-1.90]	1.29	[0.90-1.85]
Diarrhea	1.01	[0.66-1.53]	0.89	[0.54-1.48]
Sore throat	0.87	[0.51-1.48]	0.78	[0.43-1.43]
Headache	0.85	[0.62-1.16]	0.89	[0.62-1.28]
Nausea and or vomiting	0.81	[0.48-1.37]	0.68	[0.34-1.38]
Sleep disturbance	0.81	[0.46-1.43]	0.73	[0.34-1.56]
Chest Pain or discomfort	0.65	[0.47-0.91]	0.5	[0.32-0.77]
Dizziness	0.64	[0.38-1.07]	0.51	[0.25-1.04]
Emotional Disturbance	0.59	[0.48-0.73]	0.64	[0.49-0.84]
Abdominal pain	0.5	[0.37-0.67]	0.43	[0.29-0.62]
Arthralgia	0.46	[0.33-0.64]	0.39	[0.25-0.59]
Myalgia	0.46	[0.37-0.57]	0.44	[0.34-0.57]
Dyspnea and or Shortness of breath	0.45	[0.26-0.78]	0.57	[0.31-1.04]

Supplementary Table 7:Hazard ratios for each of the EHR-captured symptom, calculated by Cox proportional hazards models, and adjusted for age, gender, presence of a chronic medical condition and time (number of days since study initiation) are presented to account for the time from symptoms onset to COVID-19 testing results
