# Supplementary material for: Longitudinal symptom dynamics of COVID-19 infection
Source: Nat Commun. 2020 Dec 4;11:6208. doi: 10.1038/s41467-020-20053-y (PMC7718370; doi:10.1038/s41467-020-20053-y)
Supplement: Supplementary file 3 — Reporting Summary [file 41467_2020_20053_MOESM3_ESM.pdf]

nature research

Corresponding author(s): Prof. Eran Segal

Last updated by author(s): Oct 29, 2020

Reporting Summary

Nature Research wishes to improve the reproducibility of the work that we publish. This form provides structure for consistency and transparency in reporting. For further information on Nature Research policies, see our Editorial Policies and the Editorial Policy Checklist.

Statistics

For all statistical analyses, confirm that the following items are present in the figure legend, table legend, main text, or Methods section.

n/a	Confirmed	
☐	☒	The exact sample size (n) for each experimental group/condition, given as a discrete number and unit of measurement
☐	☒	A statement on whether measurements were taken from distinct samples or whether the same sample was measured repeatedly
☐	☒	The statistical test(s) used AND whether they are one- or two-sided<br>*Only common tests should be described solely by name; describe more complex techniques in the Methods section.*
☐	☒	A description of all covariates tested
☐	☒	A description of any assumptions or corrections, such as tests of normality and adjustment for multiple comparisons
☐	☒	A full description of the statistical parameters including central tendency (e.g. means) or other basic estimates (e.g. regression coefficient) AND variation (e.g. standard deviation) or associated estimates of uncertainty (e.g. confidence intervals)
☐	☒	For null hypothesis testing, the test statistic (e.g. F, t, r) with confidence intervals, effect sizes, degrees of freedom and P value noted<br>*Give P values as exact values whenever suitable.*
☒	☐	For Bayesian analysis, information on the choice of priors and Markov chain Monte Carlo settings
☒	☐	For hierarchical and complex designs, identification of the appropriate level for tests and full reporting of outcomes
☒	☐	Estimates of effect sizes (e.g. Cohen's d, Pearson's r), indicating how they were calculated

Our web collection on statistics for biologists contains articles on many of the points above.

Software and code

Policy information about availability of computer code

Data collection	Retrospective Electronic health records data and longitudinal self reported symptoms originates from Maccabi Health Services
Data analysis	Data were analyzed using C++ and Python 3.5 , Code is available at https://github.com/barakm-ki/Symptoms-dynamics-of-COVID-19-infection

For manuscripts utilizing custom algorithms or software that are central to the research but not yet described in published literature, software must be made available to editors and reviewers. We strongly encourage code deposition in a community repository (e.g. GitHub). See the Nature Research guidelines for submitting code & software for further information.

Data

Policy information about availability of data

All manuscripts must include a data availability statement. This statement should provide the following information, where applicable:

- Accession codes, unique identifiers, or web links for publicly available datasets
- A list of figures that have associated raw data
- A description of any restrictions on data availability

The data that support the findings of this study originate from Maccabi Health Services. Restrictions apply to the availability of these data and they are therefore not publicly available. Due to restrictions, these data can be accessed only by request to the authors and/or Maccabi Health Services

Field-specific reporting

Please select the one below that is the best fit for your research. If you are not sure, read the appropriate sections before making your selection.

☒ Life sciences ☐ Behavioural & social sciences ☐ Ecological, evolutionary & environmental sciences

For a reference copy of the document with all sections, see nature.com/documents/nr-reporting-summary-flat.pdf

Life sciences study design

All studies must disclose on these points even when the disclosure is negative.

Sample size	No sample size calculation was preformed since sample size was determined by the number of patients identified in the electronic health records and self-reported symptoms surveys
Data exclusions	In the analysis we used self-reported symptoms surveys ,(3.9%) of the surveys were not fully completed by participants and were excluded
Replication	N/A (this was an observational study describing the characteristics of patients with and without COVID-19)
Randomization	Randomization was not applicable in this study since we analysed existing retrospective Electronic health records and self-reported symptoms surveys
Blinding	Blinding was not applicable in this study since we analyzed existing anonymized retrospective Electronic health records and self-reported symptoms surveys

Reporting for specific materials, systems and methods

We require information from authors about some types of materials, experimental systems and methods used in many studies. Here, indicate whether each material, system or method listed is relevant to your study. If you are not sure if a list item applies to your research, read the appropriate section before selecting a response.

Materials & experimental systems

n/a	Involved in the study	
☒	☐	Antibodies
☒	☐	Eukaryotic cell lines
☒	☐	Palaeontology and archaeology
☒	☐	Animals and other organisms
☐	☒	Human research participants
☒	☐	Clinical data
☒	☐	Dual use research of concern

Methods

n/a	Involved in the study	
☒	☐	ChIP-seq
☒	☐	Flow cytometry
☒	☐	MRI-based neuroimaging

Human research participants

Policy information about [studies involving human research participants]

Population characteristics	We included a total of 206,377 individual who had information on symptoms from either surveys or primary care visits, including 2,471 who tested positive for COVID-19.<br>Population characteristics included : age, gender and past chronic medical condition.
Recruitment	No recruitment
Ethics oversight	The study protocol was approved by Maccabi Health Services' institutional review board

Note that full information on the approval of the study protocol must also be provided in the manuscript.
